# Contrasting seasonal responses to wind in migrating songbirds on a globally important flyway

**DOI:** 10.1098/rspb.2024.0875

**Published:** 2024-07-17

**Authors:** Inbal Schekler, Yoav Levi, Nir Sapir

**Affiliations:** ^1^ Department of Evolutionary and Environmental Biology and Institute of Evolution, University of Haifa, Haifa 3498838, Israel; ^2^ Israel Meteorological Service, Bet Dagan, Israel

**Keywords:** contrasting seasonal responses, Levant, nocturnal bird migration, radar ornithology, weather selectivity, wind effects

## Abstract

During spring migration, nocturnal migrants attempt to minimize their travel time to reach their breeding grounds early. However, how they behave and respond to unfavourable conditions during their springtime travels is much less understood. In this study, we reveal the effects of atmospheric factors on nocturnal bird migration under adverse conditions during spring and autumn, based on one of the most detailed bird migration studies globally, using radar data from 13 deployments over a period of seven years (2014–2020) in the Levant region. Using ERA5 reanalysis data, we found that migratory birds maintain similar ground speeds in both autumn and spring migrations, but during spring, when encountering unfavourable winds, they put more effort into maintaining their travel speed by increasing self-powered airspeed by 18%. Moreover, we report for the first time that spring migrants showed less selectivity to wind conditions and migrated even under unfavourable headwind and crosswind conditions. Interestingly, we discovered that temperature was the most important weather parameter, such that warm weather substantially increased migration intensities in both seasons. Our results enhance our understanding of bird migration over the Levant region, one of the world’s largest and most important migration flyways, and the factors controlling it. This information is essential for predicting bird migration, which—especially under the ongoing anthropogenic changes—is of high importance.

## 1. Background

Billions of birds migrate hundreds or thousands of kilometres between distant breeding and wintering grounds, during which they encounter various environmental conditions to which they need to respond properly to survive and reproduce. To accomplish their long migration flight, migrating birds evolved different mechanisms of sensing and responding to the environment [1–3], and it has been suggested that different selection pressures in each migration season could affect bird behaviour during the journey. Spring nocturnal migrants are expected to minimize the duration of their journey [4–6] since birds arriving early at the breeding grounds may improve their reproductive output by, for example, occupying higher-quality territories [7,8]. Following the autumn migration, there is presumably less competition for wintering territories, allowing the birds to minimize the energetic cost of the travel and to consider time economy to a lesser extent. The consequences of adopting a time-minimizing strategy in the spring and energy-minimizing behaviour during autumn are reflected in the migration flight speeds of the nocturnal migrants [9], with spring nocturnal migration being usually faster than autumn migration [6,10–12]. This difference is also evident in the duration of stopovers, which is expected to have an even stronger impact on the total migration speed [13]. Although there are examples of species that migrate faster in autumn than in spring [6], such as the white-fronted goose (*Anser albifrons*) [14], white stork (*Ciconia ciconia*) [15] and booted eagle (*Hieraaetus pennatus*) [16], passerines, which comprise the majority of the nocturnal migrants, migrate faster in spring [6,10].

While differences in migration speed between the two seasons are generally well documented [6,10,11,17–19], the impacts of specific meteorological factors when deciding to commence and maintain flight are much less understood. Specifically, the response of the migrants under unfavourable conditions during the spring, just before the breeding period, is largely understudied. Horton *et al.* [17], for example, showed that in the northeastern continental USA, birds migrate faster during the spring, having both higher airspeed and groundspeed. In addition, spring migrants were found to compensate more for wind drift compared with autumn migrants. Nussbaumer *et al.* [20] argued that the difference in groundspeed between the seasons (higher in spring than in the autumn) is mainly owing to differences in wind support and is not owing to differences in bird airspeed (refer also [18]). In the only study published so far from the Levant—an area in the Eastern Mediterranean region of West Asia—Liechti & Bruderer [19] showed that in southern Israel, migrant groundspeed is lower while airspeed is slightly higher during the spring than during the autumn. These studies, which have provided mixed evidence, emphasize the need to enhance our basic knowledge regarding bird responses to various wind conditions and specifically to the ways birds select the conditions under which they migrate. In regions outside Europe and the USA, where almost all previous studies in this field have taken place, different wind conditions may govern spring migration. In addition, the migrants may face distinct ecological pressures owing to the longer distance from their breeding grounds and might also encounter challenges stemming from different geographic (including topographic) features. Therefore, exploring these considerations in regions outside Europe and North America can help shed light on the mechanisms shaping bird migration under a wider variety of environmental conditions.

Several meteorological factors have been found to affect migrating birds, with wind being one of the most important variables affecting in-flight behaviour [2,21–24]. Wind exerts a multifaceted influence on bird migration, impacting factors such as time considerations, energy expenditure and overall migration strategy [22,25]. The wind may alter flight paths and induce route adjustments, jeopardize flight stability and influence departure and landing decisions, as well as in-flight altitude selection [2]. Generally, birds will prefer migrating with following and calm winds, with no or weak crosswinds, and they will tend to avoid flying in headwinds [21,22]. Temperature is also known to be among the most important atmospheric factors affecting migration flight timing [26–28]. It is generally accepted that increased temperature in spring promotes departure and boosts migration intensities [21,22,27]. This behaviour might be driven by the birds' need to acquire information regarding the environmental conditions prevailing along their migration route and at their breeding grounds, and owing to spatial correlation in these conditions over large scales [29]. Knowledge of local conditions could predict the conditions further along their journey and at their destination [30–33]. Much less is known about temperature effects in the autumn [34], but previous studies have typically shown a tendency to migrate on days with cooler temperature [22]. Other weather parameters found to affect migration flights are precipitation, which inhibits take-off, induces termination of migratory flight and generally decreases migration intensity [22,24,35,36]. Humidity, while usually not a key factor affecting migration intensity, can have a negative effect in some cases, mainly during the autumn [22,37].

The Palearctic–Afrotropical-bird migration system is the largest landbird migration network in the world, with more than 2 billion individuals travelling annually [11,38]. In an attempt to avoid flying over it or to minimize its crossing distance, migrants are funnelled through both the westernmost and the easternmost edges of the Mediterranean Sea [39], which is a prominent ecological barrier within this migration flyway. The Levant region in the Eastern Mediterranean, including Israel, constitutes a land bridge located at the heart of this globally important migration flyway between Eurasia and Africa [11]. Consequently, this region is characterized by high diversity and abundance of migrating birds [40]. Despite its importance, no systematic studies of bird migration have taken place in this region, with the exception of an intense radar research project during 1991–1992 during which bird migration data were collected in two localities in southern Israel, which substantially contributed to our understanding of bird migration in this region [19,41]. The meteorological conditions in this region are unique, particularly during the spring season, when adverse conditions including strong headwinds and crosswinds dominate the airspace through which migrants travel. The global pattern, which is shared across Europe and North America (where most migration studies have taken place so far), is of westerly winds blowing from southwest to northeast, creating favourable conditions for migrants during spring. Conversely in Israel, where there is approximately 200 km-ong north–south coastline bordering the eastern Mediterranean Sea, weather conditions are strongly influenced by sea breeze [42,43], blowing from the northwest in both seasons. This results in challenging conditions, with headwinds during spring and predominantly favourable tailwind conditions during autumn. These unique conditions offer an exceptional opportunity to investigate migration hypotheses concerning the influence of meteorological conditions on migration under unfavourable and favourable conditions.

In this study, our goals were to reveal the atmospheric factors affecting nocturnal migration in Israel and to explore potential differences in the selectivity of specific meteorological conditions between the seasons using comprehensive data that were collected by 10 dedicated bird radar deployments and three meteorological radars over the course of 7 years, possibly comprising one of the most detailed studies of bird migration in any country to date. Analysing how migrating birds respond to environmental factors is crucial for understanding migration mechanisms and the factors affecting them. Furthermore, it facilitates the forecasting of migration intensity, allowing us to make better use of the services that migratory birds provide, mitigate human–bird conflicts and provide critical information to aid their conservation [28,44–47].

Following the findings of previous studies, we specifically predict that: (i) tailwind will have the most important and positive effect on migration intensity and conversely headwind will have a negative effect, as has been reported in Europe [2,36,48]; (ii) crosswind will have a negative effect on migration intensity [22]; (iii) eastern winds, blowing towards the Mediterranean Sea, will have a more substantial negative effect on migration intensity than winds blowing towards the east because birds will try to avoid drifting over the sea [49]; (iv) the effects of temperature on migration intensity will be positive in the spring [26,27] and negative in the autumn [21,22]; and (v) between-season differences in bird migration speed will include higher groundspeed in the autumn and higher airspeed in the spring, as reported earlier in this region [19].

To study migrants’ in-flight behaviour, we used a large amount of data, including a total of 3651 migration nights from 13 different locations in Israel, including weather radars (3 locations, a total of 1688 migration nights) and vertical-looking radar deployments (10 locations, 1963 migration nights) during the years 2014–2020.

## 2. Methods

### (a) Radar data collection and processing

**Figure 1.**
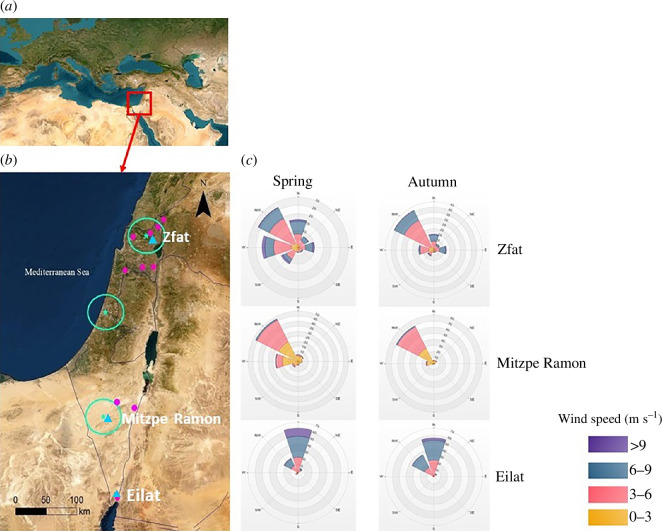
(*a*) Satellite imagery of the broader geographic region of the study area with the study area highlighted by a red square. (*b*) The weather radars (green stars), their 25 km radius coverage (green circles), vertical-looking radars (pink dots), wind sensors (blue triangles) and their location names and international borders (black lines). For detailed information, please see table 1 in the electronic supplementary material. (*c*) Wind roses based on wind sensors at heights of 10–45 m above ground level (AGL) in three locations: Eilat (the southernmost triangle), Mitzpe Ramon and Zfat (the northmost triangle) in spring and autumn.

We used data from two types of radars: three stationary single-polarization weather radars and four mobile BirdScan-MR1 vertical-looking radars that were positioned at 10 different locations across Israel (figure 1*a*, electronic supporting material, figure 1). In general, there are two basic flight strategies of migratory birds over land. The first is by flapping, which is done mainly by small birds such as most passerines and waders [11,48], primarily during the night. These birds tend to spread across the migration flyway and may commonly migrate over the sea. The second is by soaring-gliding flight, which is done mainly by large species with a relatively large wing surface area relative to their body mass, allowing them to use rising air currents during migration while saving energy [48,50]. These large birds migrate primarily during the day when updrafts are available and often do so in concentrated streams where topography favours the development of updrafts [11]. While automatic algorithms have been recently developed and applied in weather radars for passerine migration [35,51], resulting in significant progress in our understanding of this phenomenon [36,52–56], there is currently only one algorithm available for the automatic detection of soaring bird flocks in weather radars [57], which does not yet provide migration densities. Owing to this gap in knowledge and the distinct mechanisms of migration between diurnal and nocturnal migration, our study focuses on investigating the patterns of nocturnal migration, constituting a much higher densities of migrants [11] and over 70% of the migrating birds in the Levant region [40].

*Weather radars*: We obtained the raw data from a weather radar operated by the Israel Meteorological Service (IMS) in Bet Dagan in Central Israel and from two radars operated by the meteorology unit of the Israeli Airforce at Meron (MER) and Mitzpe Ramon (RAM) in the north and south of the country, respectively. Weather radar data were collected during spring and autumn migration seasons between the years 2014 and 2018, with the exception of autumn data from 2016 and 2017 that were missing from the MER and RAM archives. Additionally, the RAM data in years prior to 2017 were too sparse to interpret bird movement and were consequently excluded. We were thus able to use data from five springs and five autumns for IMS, five springs and three autumns for MER and two springs and one autumn for RAM (electronic supplementary material, table S1). The weather radars emitted C-band electromagnetic waves and recorded returned signals, known as reflectivity, from objects in the atmosphere at a polar resolution of 125 m in range and 1° in azimuth. The radars scanned the atmosphere at varying tilt angles, and from their volume data, we created vertical profiles of migration traffic rate (MTR; birds km^−1^ h^−1^) up to an elevation of 1500 m above the ground and within a 25 km range from the radar following procedures described by Dokter *et al.* [35], using the ‘vol2bird’ package in R [58].

MTR is defined as the number of birds that passed a theoretical 1 km transect perpendicular to the direction of movement over 1 hour. The analysis included data collected between sunset and sunrise, and the MTR was summed over all elevation bins and averaged for each night.

*Vertical-looking radars*: Data were collected using four vertical-looking X-band radars (BirdScan MR1, manufacturer: Swiss Birdradar Solution AG, Winterthur, Switzerland) that were positioned at 10 different locations across Israel between 2015 and 2020 (electronic supplementary material, table S1). Most of the radars were operated for one spring and one autumn season, with the exception of the Hula Valley radar that collected data for two springs and two autumns. The BirdScan radars were operated in several modes: static short-pulse (65 ns, range resolution 7.5 m, pulse repetition frequency (PRF) 1800 Hz), static long-pulse (750 ns, range resolution 110 m, PRF 785 Hz) and rotating short-pulse and rotating long-pulse. When the antenna rotated, it provided information about flight direction and flight speed, and therefore we only used data generated under these settings. For data collected during the short-pulse mode, we only considered readings at 50−800 m AGL because the maximal detection range of small birds under short-pulse does not exceed 800 m AGL [59] and because the radar cannot properly detect targets below 50 m AGL. For data from the long-pulse setting, we included readings from elevations of 800−1500 m AGL. The BirdScan radar automatically classifies echoes by the characteristics of the echo signature—including the wing-beat pattern—to bird or non-bird targets, and further classifies birds into several categories, including passerine-type, wader-type and flock [60]. In addition, to exclude rain clutter, we downloaded dates and times of rain events for each radar location from the IMS website (https://ims.gov.il/he/data_gov), visualized relevant rain events for each radar and excluded these events from the data. As with weather radars, we included only data between sunset and sunrise, and the MTR was calculated, summed over all elevation bins and averaged for each night. For removing local, non-directional movements from our data, we followed Shi *et al.* [61] and calculated the proportion of directional movements per night by applying the Rayleigh test on the directions of bird echoes. Accordingly, we used only the directional proportion of MTR for each night.

In addition to MTR, we extracted the mean groundspeed per night and the mean direction of migration per season from each vertical-looking radar, from nights with at least five available bird tracks recorded by the radar. In total, we analyzed 3651 nights of migration from 13 different locations of weather radars and vertical-looking radars.

### (b) Modelling bird intensities

From previous work [20,23,27,36,37], we identified several environmental factors that may affect bird densities and obtained datasets for their use as covariates in our models. These included several weather variables, geographic variables and a corrective variable. *Weather variables*: we download ERA5 reanalysis data [62] at the maximal resolution (hourly, 0.25° × 0.25° and pressure levels: 1000–550 hPa) for the years 2014–2020 for spring (March–May) and autumn (August–November). We extracted the following variables from the ERA5 reanalysis: (1) temperature (°C); (2) the east–west wind speed component *u* (m s^−1^), defined here as being positive for eastward winds; (3) the north–south wind speed component *v* (m s^−1^), defined here as being positive in the direction of migration (north in the spring and south in the autumn) to facilitate a simple between-season comparison; (4) relative humidity (%; the water vapour pressure as a percentage of the value at which the air becomes saturated); (5) vertical velocity (Pa s^−1^; the speed of air motion in the upward or downward direction); and (6) cloud cover (dimensionless; the proportion of a grid box covered by clouds that varies between 0 and 1). In addition, we calculated (7) the overall wind speed s (m s^−1^) using: $=\sqrt{u^{2}+v^{2}}$ .

We calculated the mean of each variable for each night and used the geopotential height to interpolate the data to a level of 500 m above the elevation of each radar. In addition, we calculated the change relative to the preceding night’s (8) temperature, (9) east–west component of the wind and (10) north–south component of the wind. We used the geographic variables (11) latitude (12) longitude and (13) elevation and added (14) the ordinal date as well. Compared with the vertical-looking radars, weather radars may detect lower rates of MTR, especially in low elevations [63]. In addition, both RAM and MER weather radars are located at relatively high elevations (868 m and 1214 m above sea level (ASL), respectively) and the radars are positioned high above their surrounding areas. Positioning weather radars at high elevations is a common practice as a result of the need to minimize beam blockage owing to topography and man-made structures. This, nonetheless, may cause the detection of only a small part of the migration, as demonstrated in MER radar that recorded only 10% of the MTR recorded by a vertical-looking radar located just 19 km from it but 800 m below it [64]. Therefore, to combine data from weather and vertical-looking radars, we added the radar type (15) as a corrective variable and expected the BirdScan radars to detect higher MTR. Following the methodology employed in numerous other research studies [23,65,66], and acknowledging the distinct characteristics of the two migration seasons in terms of timing, bird composition, migration routes and meteorological parameters, we conducted separate analyses for the spring and autumn datasets. The log of the average nighttime MTR+1 (for meeting the assumptions of normality) was the response variable, with a total of 15 predictor variables (ten weather covariates, three geographical, one corrective and the ordinal date). Pairwise Pearson correlations among predictors were examined and were found to be <0.7 and generally low and therefore all of the covariates were used in the model. To model the relationships between MTR and the covariates, we used the boosted regression trees (BRT) model with the gbm.step function in the R package ‘dismo’ [67]. The models had a Gaussian error distribution, and after running a grid search to tune the hyperparameters, we used a 10-fold cross-validation, a tree complexity of 10, a learning rate of 0.001, a bag fraction of 0.5 and a minimum of 25 trees per step.

### (c) Wind and speed data analysis

To estimate the birds’ airspeed, we calculated the wind direction (where the wind is blowing from) using

$\alpha_{wind}=atan2\;\left(u,v\right),$ and tailwind speed using

,$$TW=\;s\;\times \cos\left(\alpha_{wind}-\;\alpha_{migration}\right)$$

where *TW* is the average tailwind speed per night and location, $\alpha_{wind}$ is the wind direction and $\alpha_{migration}$ is the mean migration direction for each season in each site. Airspeed was then calculated by subtracting the tailwind component from the birds’ groundspeed. Using the R package ‘lmerTest’ [68], we applied linear mixed models (LMM) to test for seasonal differences in groundspeeds and airspeeds, with radar location as a random factor. We conducted independent samples *t*-tests to compare four meteorological variables: temperature, *u* component of the wind, *v* component of the wind and overall wind speed between the spring and autumn seasons. To account for multiple comparisons, we applied the Bonferroni correction and adjusted the significance level to 0.0125. All analyses were done in R v. 4.0.3 (R Core Team) [69]. To validate and explore the wind patterns in the general areas of the study, we also collected data on wind intensity and direction from standard wind sensors (Young wind monitor 05103, https://www.youngusa.com) deployed by the IMS during 2012–2022. The sensors were located 10–45 m AGL in three locations across Israel that are close to many of the radars from which the data were collected (figure 1*a*).

## 3. Results

### (a) Factors affecting migration intensity

**Figure 2.**
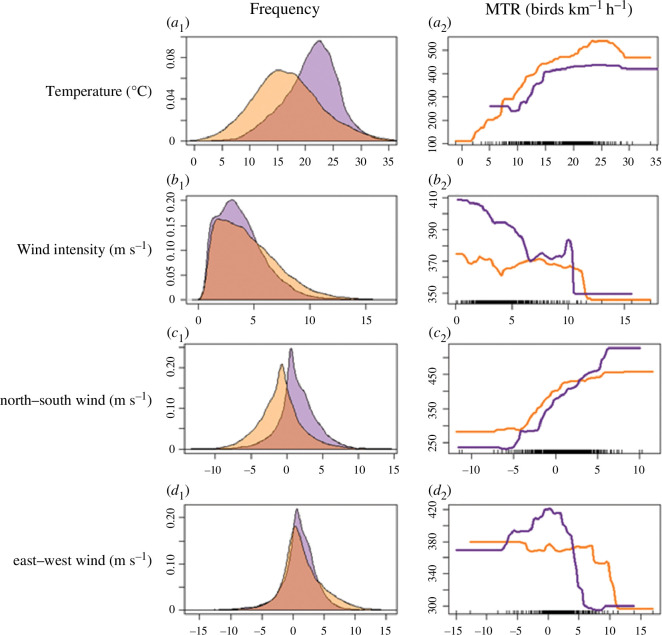
*Left*: Weather covariate histograms in autumn (purple) and spring (orange) of temperature (*a_1_*; °C), overall wind speed (*b_1_*; m s^−1^), north–south wind speed (*c_1_*; m s^−1^) and east–west wind speed (*d_1_*; m s^−1^). *Right*: Marginal responses of MTR (birds km^−1^ h^−1^) based on BST models in relation to temperature (*a_2_*), overall wind speed (*b_2_*), north–south wind speed (*c_2_*) and east–west wind speed (*d_2_*).

**Figure 3.**
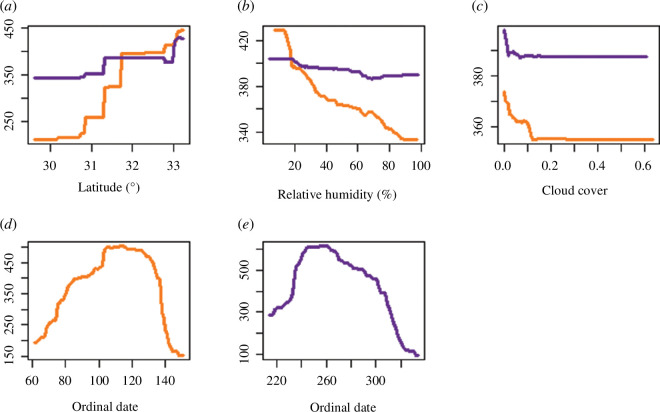
Marginal responses of MTR (birds km^−1^ h^−1^) based on BST models in relation to latitude (*a*), relative humidity (*b*), cloud cover (*c*) and ordinal date (*d,e*) in spring (orange) and autumn (purple).

The temperature and the date were the two most important covariates affecting the MTR in both spring and autumn models (except for the radar type covariate, which was a corrective variable; table 1). Yet, their importance was not of the same order in the two seasons. In the spring, the most important variable was temperature, followed by the date, with a substantial decrease in the relative importance of the next most important variable, which was latitude. In autumn, the date was the most important variable with a relative importance almost three times greater than temperature. Birds preferred migrating on warmer days in both seasons (figure 2*a* _2_). In autumn, the MTR increased up to a threshold of about 15°C while in spring, MTR increased with temperature up to about 25°C. Despite the fact that the mean temperature in the spring (16.71°C) was significantly lower than in the autumn (21°C; *p* < 0.001, *t*_77165_ = 122.08) with fewer days with temperatures higher than 15°C (figure 2*a* _1_). There is a peak in the migration intensities around mid-April during the spring season and at the outset of September in the autumn (figure 3*d*,*e*).

**Table 1.** The importance of the covariates in spring and autumn as determined by BST models. The relative importance of covariates is based on the number of times a variable is selected for 751 splitting, weighted by the squared improvement of the model, averaged over all trees and scaled 752 so that the sum adds to 100 [58].

	importance rank		relative importance	
	spring	autumn	spring	autumn
radar type (weather/vertical-looking)	1	1	51.99	66.01
temperature (°C)	2	3	14.83	5.55
ordinal date	3	2	11.29	14.86
latitude	4	7	5.80	1.11
north–south wind speed (m s^−1^)	5	4	3.95	3.42
relative humidity (%)	6	14	2.14	0.55
temperature difference (°C)	7	10	1.65	0.66
east–west wind speed (m s^−1^)	8	12	1.61	0.58
north–south wind speed difference (m s^−1^)	9	13	1.47	0.56
wind intensity (m s^−1^)	10	8	1.07	0.85
elevation	11	5	1.06	2.61
east–west wind speed difference (m s^−1^)	12	9	1.03	0.72
vertical velocity (Pa s^−1^)	13	12	0.95	0.57
longitude	14	11	0.64	0.58
cloud cover	15	15	0.50	0.27

Out of the three wind components used in the model (overall wind speed, the north–south component of the wind speed and the east–west component of the wind speed), the north–south component of the wind speed had the highest relative importance in both spring and autumn, with higher MTR values in north–south winds in the general directions of the migration and lower migration intensity under headwind, as expected. Latitude had a larger influence in the spring, and in both seasons, higher migration intensities were found in more northern locations. Relative humidity and cloud cover had negative effects on bird MTR in both seasons (figure 3) but were not key factors affecting migration intensity. The change relative to the preceding night in all of the tested weather parameters (temperature, north–south wind and east–west winds) was generally not of high importance and affected MTR much less than the corresponding night-of-migration parameters. The models had cross-validation correlations of 0.77 in the spring and 0.88 in the autumn.

### (b) Migration speed and wind selectivity

Bird mean groundspeed was higher in the autumn than in the spring (*p* = 0.009, *t* = −2.58, d.f. = 1854.13) but with only a small absolute difference of 4% (0.6 m s^−1^). In contrast, the mean airspeed was 18% lower in the autumn than in the spring (*p* < 0.001, *t* = 19.87, d.f. = 1854.26), with a difference of 2.5 m s^−1^ (table 2). Wind direction during the two migration seasons was generally from the north to north-west across Israel (figure 1*b*), and overall wind speed was significantly higher in the spring ( figures 1b and 2b). Consequently, wind patterns were overall supportive of migration in autumn, while spring migrants primarily experienced headwinds when flying over Israel. The higher spring airspeed of migrating birds allowed them to fly at nearly identical groundspeeds in both seasons.

**Table 2.** Mean and s.d. of groundspeed and airspeed (m s^−1^) for spring and autumn from 10 deployments of vertical-looking radars across Israel.

	groundspeed (m s^−1^)		airspeed (m s^−1^)	
	mean	s.d.	mean	s.d.
spring	15.1	3.27	16.06	4.4
autumn	15.7	3.58	13.6	3.72

Notably, bird migration intensity decreased dramatically above a wind speed of 10 m s^−1^ in both seasons, and bird MTR was related to wind support, with lower migration intensities in headwind conditions and higher migration intensities in tailwind conditions in both seasons. Overall, the birds were more selective of wind conditions in the autumn. The higher selectivity of the migrants in this season was manifested by higher densities under preferable tailwinds and lower intensities under headwinds, compared with the spring (figure s 1 *b* and 2*c* _1_), during which birds continued migrating even if conditions seemed to be sub-optimal (figure 2*c* _2_). In addition, during the autumn, birds preferentially migrated under lower wind speeds (figure 2*b* _2_). Similar relationships were also found for crosswinds (east–west winds), which hindered migration but not to the same extent in the two migration seasons, as birds were less selective during spring. In this season, birds flew regardless of crosswind speed, also up to a wind speed threshold of about 10 m s^−1^, while during the autumn, birds preferred migrating only when no strong crosswinds prevailed such that the overall range of crosswind speeds during which migration took place was substantially narrower than in spring. Interestingly, migration steeply decreased in cross-wind speeds above 5 m s^−1^ to the east during autumn, and generally, in both seasons, birds preferred migrating under westward winds.

## 4. Discussion

We examined the factors affecting nocturnal bird migration in the two migration seasons using a large dataset of two radar types from 13 different locations in Israel, comprising the first study of its kind in the Levant region, which sits at the core of a globally important migration flyway [11], renowned for its rich diversity and abundance of migrating birds [40]. Additionally, this region is distinguished by unique weather conditions and, consequently, we explored the effects of weather on bird migration intensity as no previous data are available regarding this. We found strong effects of specific weather parameters and substantial differences in the in-flight selective behaviour in relation to the wind between spring and autumn migrations. Birds showed more selectivity during autumn when choosing under which wind conditions to migrate with respect to all three components of the wind: tailwind, crosswind and overall wind speed. In addition, the birds’ groundspeed was slightly higher in the autumn but their airspeed was 18% higher in the spring, suggesting that the birds put a lot more effort into migrating during the spring and behave more like time minimizers when encountering sub-optimal wind conditions during this season. The ratio between the mean airspeed of the birds in the two seasons reported here is similar to those reported elsewhere [6,10,17], but it specifically contrasts with estimates from Europe that reported relatively constant airspeed between the two seasons [18,20]. The groundspeed ratio is similar only to the single other study carried out in this region [19] and it contrasts with the findings of many other studies that reported higher groundspeed during spring migration [6,10,17,18,20].

We found that in the Levant region, in contrast to findings from other studies performed in Europe [2,24,36], temperature played the most important role among all weather variables in determining bird migration intensity in both seasons. Notably, the effect of temperature on bird migration intensity during autumn was different from our hypothesis and contrasted with the findings of earlier studies [22], with higher bird MTR during warmer days. As demonstrated in recent studies [27], the progress of the season, represented by the ordinal date, had a strong effect on migration intensities, with a bell-shaped pattern of migration. MTR increased with latitude during both seasons although to a much steeper degree during spring, possibly influenced by spatial patterns in the quality of stopover sites. Israel’s diverse ecoregions, spanning from harsh desert in the south to semi-arid regions and Mediterranean scrub in the north (figure 1), are probably responsible for this trend. The higher densities of migrants observed in northern latitudes may be attributed to more favourable conditions for stopping over in both seasons.

Migrating birds must balance time and energy considerations when making behavioural decisions during migration, including during stopover and flight [70]. Flight initiation and continuation under specific weather conditions have energy and time consequences, with implications for the accomplishment of the migration journey and the survival and reproduction of the birds in the following seasons. Although waiting for favourable wind conditions, as documented in Europe [18,20], may improve birds' energy balance of owing to lower in-flight metabolic expenditures, there could also be a substantial cost of waiting for favourable wind conditions, as it may greatly draw out the overall journey [70,71]. In the spring, when there is an advantage of prompt arrival to the breeding grounds [7], passerines may migrate faster to complete their journey earlier [6,17,22]. The variation in age composition between seasons, with a higher proportion of juveniles in autumn, may partially explain differences in migrant behaviour between seasons. However, this phenomenon was observed particularly in raptor migration [72,73] and is less pronounced in the case of passerine migration [74,75]. We showed that in the Levant region, where tailwind conditions are common during the autumn and rare during the spring, nocturnal birds probably expend more energy during spring migration by flying considerably faster in the air. In addition, the birds show less selectivity for wind conditions during this season and migrated even under unfavourable conditions, probably to complete their migration faster at the expense of higher metabolic expenditure.

Further support for season-dependent selectivity is found in the relationships between MTR and other wind variables. Under very similar crosswind conditions in both seasons, we found selectivity for slower crosswind during the autumn migration compared with the spring. Crosswinds may cause birds to drift off course and could incur high flight energetic costs, and they may even increase bird mortality [2,76], particularly in the case of juveniles, which are more susceptible to drifting compared with adults [25,73,77]. This between-season difference in the selection of crosswind conditions thus uncovers the importance of time-related considerations in the spring, as opposed to energy considerations that are probably more important l to birds during autumn [5]. Similarly, the birds preferred flying under weak winds during the autumn migration, while no selectivity for lower wind speed (up to a threshold of 10 m s^−1^, which is close to the birds’ own speed) was found during spring migration.

Contrary to our hypothesis and to other studies in which the wind was found to be the most important weather parameter affecting migration intensity [2,21,36], we found that among all weather variables, temperature had the greatest effect on bird MTR, especially during the spring. This has been reported elsewhere [26,27,78], but our findings further suggest that bird migration is strongly and positively affected by temperature also in autumn. In spring, one of the primary hypotheses explaining the relationship between temperature and migration is the ‘thermal wave hypothesis’ [28]. According to this hypothesis, local conditions predict the conditions further north, with seasonal patterns forming a geographical wave. For Palearctic−Afrotropical migrants in spring, we expect a wave of temperature to move from south to north [28]. During autumn, the discovery of a positive effect of temperature on migration is unprecedented as far as we know, and the thermal wave hypothesis cannot account for this relationship.

Another result, contrary to our expectation, was the effect of crosswinds on migration intensity. In general, we expected to see lower intensities of migration in strong crosswinds [22] and a preference of the migrants to fly under conditions of eastward winds (in a direction away from the Mediterranean Sea) rather than westward ones. When migrating next to the Atlantic Ocean, nocturnal migrants tried to avoid drifting over the ocean by reorienting their flights inland whereas when they were inland, the migrants showed a higher tendency to drift [79]. In the case of the Mediterranean Sea, passerine densities are highest at its western and eastern edges, where sea crossing is short or can be avoided through flight over land bridges [11,80]. These findings suggest that migrants possess awareness of their location relative to migration barriers while in flight and actively evaluate the extent for drift compensation required. These behavioural shifts could be facilitated by visual cues such as rivers and coastlines [81,82], compass directions [83,84], and possibly the interaction of various sensory systems.

We predicted that the nocturnal migrants in the Levant region would prefer flying under winds that blow towards the east, away from the sea, but we found that although there are more crosswinds towards the east both in the spring and in autumn, birds generally preferred migrating under westward winds towards the Mediterranean Sea. One possible explanation for this pattern is that the Mediterranean Sea, unlike the Atlantic Ocean, does not constitute a wide ecological barrier (and hence does not pose a serious risk) for the migrants. Many passerines migrate over the Mediterranean Sea on their way from Europe to Africa and back [39,80,85], and although they have only a few places to stop and rest during the sea crossing, it seems that they prefer drifting towards the sea than towards inland. During autumn, many of the birds migrate along the coast [86], and indeed in this season, birds preferred wind conditions that included westward winds, towards the sea, compared with the spring, which is characterized by a more inland migration [86]. It is nevertheless important to note that all of the radars, with the exception of the Bet-Dagan meteorological radar, were located tens of kilometres away from the coastline, and thus they could not document movements of migrating birds close to the sea. We hope that future deployments of radars along the eastern shoreline of the Mediterranean Sea will permit the testing of birds' coastal migration patterns in a more rigorous way.

## 5. Conclusions

Birds tended to migrate equally fast in the two migration seasons, but during the spring migration, when confronted with sub-optimal weather conditions, they invested more effort to migrate at the same pace as they migrate in autumn. Furthermore, in the spring, migrants flew even under unfavourable wind conditions and were thus less selective compared with autumn migrants. Understanding the in-flight behaviour of migrating birds is crucial for our ability to predict migration intensity at different spatial and temporal scales [27,87]. Predicting bird migration properties helps in deciphering how different environmental factors affect the birds, including their population dynamics and conservation needs, allowing the discovery of their roles in different ecosystem services. Importantly, bird migration prediction is critical for managing the implications of bird migration for human lives and the economy, in relation to bird-strike risks to aerial transportation, agriculture concerns and disease spread [44]. In addition, under the ongoing process of climate change that is already influencing the timing of different stages in the migrants’ annual cycles [88–90], forecasting future trends resulting from this change is of high importance. Our work suggests that weather conditions affect migrating birds in the Levant differently from those migrating through other regions, enhancing the understanding of the Palearctic–Afrotropical migration system and highlighting the importance of studying in-flight behavioural decisions of migrating birds in different parts of the world.

## Data Availability

The data supporting the results are archived on OSF [91]. Supplementary material is available online [92].
